# A visual analytics approach for models of heterogeneous cell populations

**DOI:** 10.1186/1687-4153-2012-4

**Published:** 2012-05-31

**Authors:** Jan Hasenauer, Julian Heinrich, Malgorzata Doszczak, Peter Scheurich, Daniel Weiskopf, Frank Allgöwer

**Affiliations:** 1 Institute for Systems Theory and Automatic Control, University of Stuttgart, Pfaffenwaldring 9, 70569 Stuttgart, Germany; 2 Visualization Research Center, University of Stuttgart, Allmandring 19, 70569 Stuttgart, Germany; 3 Institute of Cell Biology and Immunology, University of Stuttgart, Allmandring 31, 70569 Stuttgart, Germany

## Abstract

In recent years, cell population models have become increasingly common. In contrast to classic single cell models, population models allow for the study of cell-to-cell variability, a crucial phenomenon in most populations of primary cells, cancer cells, and stem cells. Unfortunately, tools for in-depth analysis of population models are still missing. This problem originates from the complexity of population models. Particularly important are methods to determine the source of heterogeneity (e.g., genetics or epigenetic differences) and to select potential (bio-)markers. We propose an analysis based on visual analytics to tackle this problem. Our approach combines parallel-coordinates plots, used for a visual assessment of the high-dimensional dependencies, and nonlinear support vector machines, for the quantification of effects. The method can be employed to study qualitative and quantitative differences among cells. To illustrate the different components, we perform a case study using the proapoptotic signal transduction pathway involved in cellular apoptosis.

## 1 Introduction

Cell populations are heterogeneous in terms of, e.g, cell age, cell cycle state, and protein abundance [1,2]. This heterogeneity is ubiquitous, even in clonal population, and influences cell fate decisions [2,3], such as cell death/proliferation [4-7]. Thus, to ultimately understand and control the behavior of populations, the key sources of cell-to-cell variability have to be unraveled. Unfortunately, this is challenging due to experimental constraints. Most experimental systems and measurement devices only allow for the simultaneous assessment of a few cellular properties on a single cell basis. This prohibits the purely experimental analysis of processes which depend on many different cellular properties. Spencer et al. [5] have shown that the experimental limitations can be overcome partially using mathematical models.

To mathematically describe heterogeneous populations, agent-based models are used most frequently. Each agent provides a mechanistic description of the signal transduction within individual cells and thus of its behavior. In such a framework, variability can be modeled by either stochastic [8-10] or deterministic [4,5,11] differences among individual cells. The source of the former is the stochasticity of biochemical reactions, while the latter may arise from genetic and epigenetic differences, environmental heterogeneity, or slow dynamic processes (such as the cell cycle).

We focus on the deterministic differences among cells — also called extrinsic factors [12] — in populations of non-interacting cells. Those differences are commonly modeled by differential parameter values and initial conditions [5,13]. Several methods exist to infer the distribution of parameters and initial conditions from experimental data [13-15] and to obtain quantitative, mechanistic models for cell populations. Unfortunately, the resulting agent-based models are in general highly complex. This complexity prevents the analysis of these models using common tools for dynamical systems [16], such as sensitivity and bifurcation analysis. To the best of our knowledge, for models of heterogeneous cell populations, no structured analysis approach is available. To study population models and to facilitate a model-driven analysis of the heterogeneity, highly flexible methods are required which do not rely on an analytical analysis.

In this work, we propose two methods to fill this gap and to facilitate the analysis of population models. These methods — *parallel-coordinates plots *[17] and *support vector (SV) machines *[18-20] — are tools widely used for the analysis of high-dimensional datasets. We outline how these tools can also be used to analyze complex models of heterogeneous cell populations, particularly addressing the question: "Which parameters cause the heterogeneity of the population's response?". Thereby, we extend our previous work [21] and consider qualitative heterogeneity among cells, in the context of cell fate decisions, as well as quantitative heterogeneity, such as the delay of a decision process.

We show that parallel-coordinates plots provide an easy tool to obtain a qualitative understanding of the system, whereas SV machines allow for assessing the performance of marker combinations quantitatively. Good markers are thereby defined as single cell parameters that facilitate a good prediction of the cell fate decision or the quantitative property under consideration of the individual cell. Furthermore, we show how the combination of parallel-coordinates plots and SV machines enables an in-depth analysis of complex models using exploration techniques.

The article is structured as follows: In the section "Methods", the considered system class and problem are described in mathematical terms, the general idea is discussed, and the application of parallel-coordinates plots and SV machines is outlined. In the section "Results", we provide an exemplary application of our method to a model of the caspase cascade. The article is summarized in the section "Discussion".

## 2 Methodology

### 2.1 Models for heterogeneous cell populations and decision processes

#### 2.1.1 Mechanistic population model

In this article, population dynamics are described using an ensemble [5,13] of cells (agents). This yields the agent-based population model:

$$\Sigma_{\text{pop}}=\left\{\Sigma \left(\theta^{\left(i\right)}\right)|i=\left\{1,\;\ldots ,\;N\right\},\;\theta^{\left(i\right)}\sim \Theta \left(\theta \right)\right\},$$

in which the superscript (*i*) specifies individual cells within the population, *N *∈ ℕ denotes the size of the cell ensemble and Σ(*θ*^(*i*)^) is the model of the *i*-th cell. The single cell model Σ(*θ*^(*i*)^) may belong to the class of Markov jump processes [15], stochastic differential equations [14], or ordinary differential equations [13]. Since in this study we are mainly interested in signal transduction and decision making, we consider ordinary differential equation models. Each individual cell of Σ_pop _is described by

$$\Sigma \left(\theta^{\left(i\right)}\right):{ẋ}^{\left(i\right)}=f\left(x^{\left(i\right)},\;\theta^{\left(i\right)}\right),\;x^{\left(i\right)}\left(0\right)=x_{0}\left(\theta^{\left(i\right)}\right),$$

with state vector $x^{\left(i\right)}\left(t\right)\in {\mathbb{R}}_{+}^{n}$ and parameter vector $\theta^{\left(i\right)}\in {\mathbb{R}}_{+}^{q}$. The vector field $f:{\mathbb{R}}_{+}^{n}\times {\mathbb{R}}_{+}^{q}\to {\mathbb{R}}^{n}$ describing the cell dynamics is locally Lipschitz and the mapping $x_{0}:{\mathbb{R}}_{+}^{q}\to {\mathbb{R}}_{+}^{n}$ is continuously differentiable. The parameters *θ*^(*i*) ^may be kinetic constants, such as synthesis, degradation, or reaction rates.

Heterogeneity among cells of the ensemble is modeled by differential parameter values *θ*^(*i*) ^and initial conditions *x*_0_(*θ*^(*i*)^) among individual cells. The density of parameters *θ*^(*i*) ^is given by a probability density function $\Theta :{\mathbb{R}}_{+}^{q}\to {\mathbb{R}}_{+}$. Thus, the probability of observing *θ*^(*i*) ^∈Ω is

$$\text{Prob}\left(\theta^{\left(i\right)}\in \Omega \right)=\underset{\Omega }{\int }\Theta \left(\theta \right)d\theta .$$

This modeling framework is highly flexible and has been proven to be very useful, especially if fast signal transduction processes, such as cellular apoptosis, are investigated. For a more detailed introduction, we refer to the work of Spencer et al. [5] and Hasenauer et al. [14]. The properties of such populations of single cells have been studied by Spencer et al. [5], while Hasenauer et al. [14] have derived a partial differential equation model for the resulting population dynamics.

#### 2.1.2 Qualitative and quantitative properties of the single cell response

Given the mathematical models introduced above, we study qualitative and quantitative properties of the single cell responses. Qualitative properties are defined as the outcome of a discrete decision processes, e.g., whether the state of a bistable system converges to one or the other stable steady state, or whether a certain concentration threshold is reached. In contrast, quantitative properties allow the assessment of small differences among cells, such as the time point when a particular threshold is exceeded.

To define single cell properties given the single cell trajectory *x*^(*i*)^(·), the functionals *F_φ_*: ℓ^1 ^→ ℝ and *F_δ_*: ℓ^1 ^→ {-1, +1} are introduced. The functional *F_φ _*is used to evaluate the quantitative property *φ*^(*i*) ^= *F_φ_*(*x*^(*i*)^(·)) ∈ ℝ, while *F_δ _*determines the qualitative property *δ*^(*i*) ^= *F_δ_*(*x*^(*i*)^(·)) ∈ {-1, +1}.

To exemplify the functionals, we consider a process in which threshold exceeding and its timing are of interest. Such processes are important, for example, in apoptotic signaling [5] and cell cycle progression [22,23], and allow for two outcomes. Either the concentration of a molecule $x_{j}^{\left(i\right)}$ within the *i*-th cell exceeds the threshold *x*_*j*, th_, *δ*^(*i*) ^= +1, or it does not, *δ*^(*i*) ^= -1. This yields the decision functional

(1) $$F_{\delta }\left(x^{\left(i\right)}\left(\cdot \right)\right):=\left\{\begin{array}{cc} +1 & \text{if }\underset{t}{\text{max }}x_{j}^{\left(i\right)}\left(t\right)\geq x_{j,\text{th}}\\ -1 & \text{otherwise}\text{.} \end{array}\right)$$

For the subgroup of cells exceeding the threshold, the time of threshold exceeding is defined by the second functional

(2) $$F_{\varphi }\left(x^{\left(i\right)}\left(\cdot \right)\right):=\text{arg }\underset{t}{\text{min}}\left\{x_{j}^{\left(i\right)}\left(t\right)\geq x_{j,\text{th}}\right\},$$

and may be employed to achieve a quantitative understanding.

Note that the response *x*^(*i*)^(·) of a cell merely depends on the cell's parameters *θ*^(*i*)^, as the single cell model is deterministic. Therefore, the quantitative and qualitative properties of a single cell can be viewed as a function of the parameters, *φ*^(*i*) ^= *φ*(*θ*^(*i*)^) and *δ*^(*i*) ^= *δ*(*θ*^(*i*)^). Differences in the parameters — as they arise between different cells — may hence influence *δ*^(*i*) ^and *φ*^(*i*)^, which determine cell fate decision and qualitative properties of the cells.

#### 2.1.3 Response markers

To understand the heterogeneity within the population response Σ_pop_, it is necessary to assess the dependency of *δ*^(*i*) ^and *φ*^(*i*) ^on the individual parameters *θ_j_*. In particular, the question arises which subset *θ***_m _**of parameters,

$$\theta_{m}:={\left[\theta_{m_{1}},\;\ldots ,\;\theta_{m_{r}}\right]}^{\text{T}},\;\text{with }m\subseteq \left\{1,\;\ldots ,\;q\right\},$$

is responsible for which aspect of the population heterogeneity. Mathematically, **m **is an index set and, e.g., for **m **= [2, 4] ^T ^only *θ***_m _**= [*θ*_2_, *θ*_4_]^T ^is considered. The question of the relative importance of different parameters directly relates to the common problem of biomarker selection for stem cells and tumor cells, which is experimentally challenging.

If there exists a subset *θ***_m _**of the parameters *θ *which allows for the reliable prediction of the response, not all sources for heterogeneity have to be assessed but only those associated to *θ***_m_**. This enables a focusing of the model development, as well as the reduction of the experimental effort.

### 2.2 Analysis of population models using data analysis tools

In this contribution, we illustrate the application of parallel-coordinates plots and support vector machines for the study of parameter dependencies and the selection of markers **m**. Parallel-coordinates plots and SV machines are well-known, but almost exclusively applied to study high-dimensional sets of measurement data. To exploit the methods for the analysis of simulation models, at first the cell ensemble is simulated for *N *≫ 1. This yields many pairs of parameters and trajectories,

$$\left(\theta^{\left(i\right)},\;x^{\left(i\right)}\left(\cdot \right)\right),\;i=1,\;\ldots ,\;N,$$

which are then used to obtain samples of quantitative,

$$S_{\varphi }=\left\{\left(\theta^{\left(1\right)},\varphi^{\left(1\right)}\right),\ldots ,\left(\theta^{\left(N\right)},\varphi^{\left(N\right)}\right)\right\},\;\text{with }\varphi^{\left(i\right)}=F_{\varphi }\left(x^{\left(i\right)}\left(\cdot \right)\right),$$

and qualitative

$$S_{\delta }=\left\{\left(\theta^{\left(1\right)},\delta^{\left(1\right)}\right),...,\left(\theta^{\left(N\right)},\delta^{\left(N\right)}\right)\right\},\;\text{with }\delta^{\left(i\right)}=F_{\delta }\left(x^{\left(i\right)}\left(\cdot \right)\right),$$

cell properties of interest. These samples contain information about the dependency of *φ *and *δ *on the parameters *θ*, being analyzed in the following. To study the high-dimensional mappings *δ *= *δ*(*θ*) and *φ *= *φ*(*θ*), parallel-coordinates plots will be employed. For the quantitative assessment of particular marker combinations SV machines will be applied. By combining both approaches it is possible to quickly gain an overview of important interrelations and quantify those.

#### 2.2.1 Combining parallel-coordinates plots and SV machines to a visual analytics system

The proposed simulation data-based analysis approach circumvents an analytical analysis of the system equations, which would be time consuming and could only be carried out by experts. However, the simulation data-based approach creates the need for analyzing the large, high-dimensional datasets, $S_{\delta }$ and $S_{\varphi }$.

**Figure 1:**
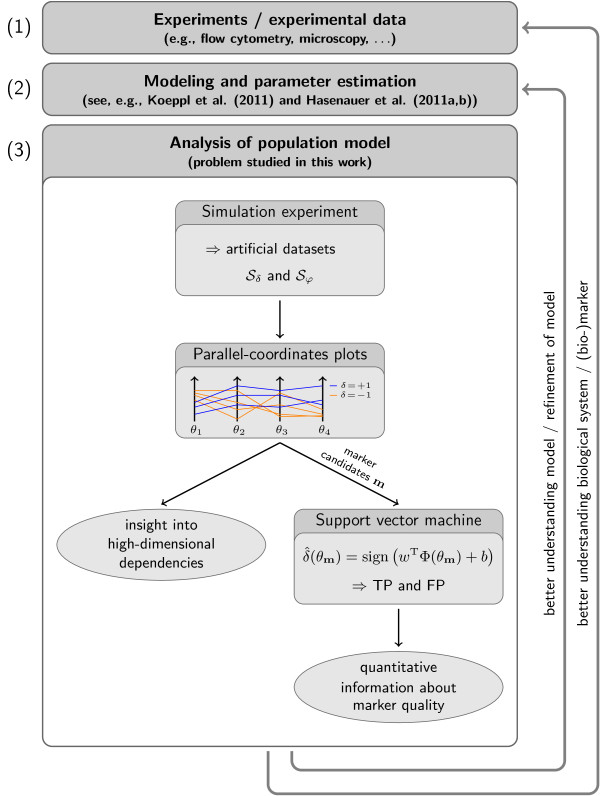
**Visual analytics for marker selection**. Illustration of the overall workflow of (1) experiments and the collection of measurement data, (2) modeling and parameter estimation, and (3) model analysis. Our visual analytics framework for marker selection in models of heterogeneous cell populations is shown in detail. Based on simulation data $S_{\delta }$ and $S_{\varphi }$ generated from the model, a visual analysis is performed using parallel-coordinates plots. Employing this visualization, insight can be gained into the dependencies of the considered properties on the parameters. Additionally, a potential marker combination **m **can be selected, here, for instance, **m **= 1 or **m **= 4, which allow for a separation of the classes. For the quantitative assessment of the informativeness of *θ***_m_**, e.g., SV classification or SV regression, may be employed.

The analysis of such datasets often relies on a reduction of complexity while preserving the important information. Visualization can help in such a situation to determine the important parameters and to avoid information loss. In this work, parallel-coordinates plots are used to gain insight into the high-dimensional dependencies and to find interesting dimensions. In this particular setting, interesting dimensions are those that clearly separate a given set of classes and thus are good candidates for the selection of potential markers **m**. In a second step, the potential markers **m **are used to train a SV machine. These SV machines allow for a quantitative evaluation of the marker quality. While SV machines are also helpful on their own, checking all possible combinations of markers would result in a combinatorial explosion. By combining SV machines and parallel-coordinates plots, the number of necessary SV machine evaluations can be decreased substantially, resulting in a tremendously reduced computational complexity. The overall workflow of the analysis illustrated in Figure 1.

Besides an improved understanding of the model, results obtained during the analysis can be used to adapt the population model or to select additional experiments. This proposed framework, integrating interactive visualization with automated methods while allowing for a feedback to the actual system/model, thus incorporates important aspects of visual analytics [24].

### 2.3 Parallel-coordinates for the analysis of high-dimensional data

**Figure 2:**
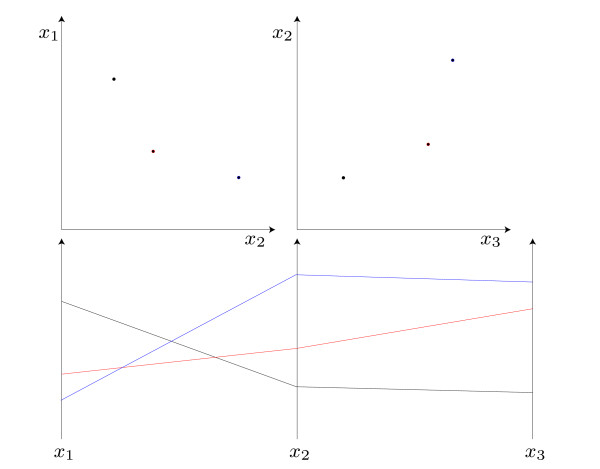
**Parallel-coordinates plots represent multi-dimensional data as polylines crossing parallel axes**. A point in Cartesian coordinates is mapped to a line in parallel-coordinates. As more axes are added, a line can be traced over all dimensions, which greatly facilitates the perception of multi-dimensional data.

Parallel-coordinates [17] are a popular visualization technique for high-dimensional data. A parallel-coordinates plot is constructed by placing axes in parallel, as illustrated in Figure 2. A single pair of adjacent axes represents a 2-D projection of the data, where a point of the corresponding Cartesian coordinates is mapped to a line in parallel-coordinates, and vice versa. Due to this point-line duality, the same patterns emerge in a parallel-coordinates plot as in the dual Cartesian coordinates. However, adding more axes not only allows to visualize a set of pairwise relations, but also supports the viewer in tracing lines over all dimensions. As a result, multi-dimensional outliers and clusters can be visualized together with 2-D relations and the distribution of values for single dimensions.

As an *N*-dimensional data point is represented by a polyline intersecting axes at the respective values, parallel-coordinates greatly suffer from overplotting if many lines have to be drawn. In the resulting clutter of lines, interesting patterns might be hidden from the user. Exploiting the point-line duality, similar clutter-reducing approaches as for Cartesian coordinates can be used, where a popular technique is to estimate the density of points (lines) and to render points (lines) transparently with blending enabled. Other approaches compute a continuous density [25] or estimate the overall density using density estimation techniques [26,27]. In this work, both alpha and additive blending is used to visualize the parameter distribution in the different classes (*φ*^(*i*) ^= 1 and *φ*^(*i*) ^= -1), enabling a qualitative analysis of their multi-dimensional shape. An example of this alpha blending is shown in the section "Results".

For the analysis of a continuous variable, colormaps can be applied to the axis representing the dependent variable *φ*^(*i*)^. Then, every polyline is rendered using a color according to *φ*^(*i*)^, such that its value can be visually determined over the whole plot. The overall distribution of colors can then be used in conjunction with the shape of lines to analyze the dependency of independent variables from the dependent. Again, overplotting can become an issue for large datasets, such that a separation in few classes and a separate visualization of those might be more informative (see example in section "Results").

### 2.4 SV machines for the quantification of marker performance

Given a basic understanding of the importance of the parameters and a potential marker combination *θ***_m_**, a quantitative assessment of the predictive power of *θ***_m _**is desirable. To achieve this, the samples $S_{\delta }$ and $S_{\varphi }$ are analyzed employing nonlinear SV classification and nonlinear SV regression, respectively. SV classification allows for the study of decision processes, while SV regression enables the analysis of quantitative system properties.

The performance of SV machines — which might be interpreted as data-based predictors — provides a measure for the quality of the marker combination *θ***_m_**. If a SV machine using only *θ***_m _**provides good predictions for a decision process which depends on *θ*, then this means that *θ***_m _**carries the most important information. This will be discussed in more detail in the following.

#### 2.4.1 SV classification

**Figure 3:**
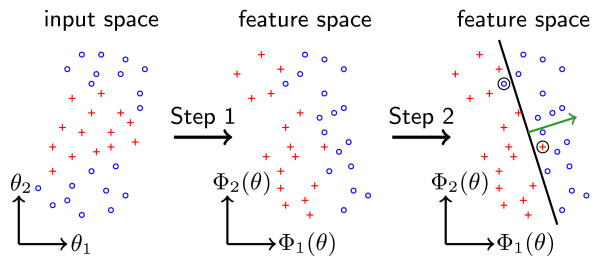
**Support vector machine for subgroup classification**. Visualization of the SV machine approach for separating cells with *δ*^(*i*) ^= +1 (+) and *δ*^(*i*) ^= -1 (◦). Left: distributed data in the input space. Middle: sample transformed in the feature space which allows for better separation. Right: separation result for separating hyperplane with normal vector *w *(→). As a perfect separation is in general not possible, misclassifications (○) exist.

The goal of the SV classification is to predict the discrete property *δ*^(*i*) ^given $\theta_{m}^{\left(i\right)}$. Thus, the nonlinear mapping *δ *= *δ*(*θ*) is approximated by the lower-dimensional nonlinear mapping $\hat{\delta }=\hat{\delta }\left(\theta_{m}\right)$. To calculate the SV classifier, a two step procedure is applied, as illustrated in Figure 3. First, a mapping $\Phi :{\mathbb{R}}^{r}\to {\mathbb{R}}^{r^{*}}$— also called kernel — is constructed that transforms the input space into a feature space of higher dimension (r* >*r*). Second, a linear separation of the data is performed in feature space [20]. Therefore, the optimization problem

(3) $$\begin{array}{c} \underset{w,b,\xi }{\text{minimize}}\;\frac{1}{2}w^{\text{T}}w+C\overset{N}{\underset{i=1}{\sum }}\xi_{i}\\ \text{subject to }\delta^{\left(i\right)}\left(w^{\text{T}}\Phi \left(\theta_{m}^{\left(i\right)}\right)+b\right)\geq 1-\xi_{i},\;i=1,\ldots ,N,\\ \;\;\;\;\;\;\;\;\;\;\;\;\xi_{i}\geq 0,\;\;\;i=1,\;\ldots ,\;N, \end{array}$$

is solved, in which *w *and *b *denote the normal vector of the separating hyperplane and its offset, respectively. The objective function combines a misclassification penalty, $\sum_{i=1}^{S}\xi_{i}$, and a margin maximization, $\frac{1}{2}w^{\text{T}}w$. The weighting of the different terms can be influenced via *C*. The constraints are that all data points $\Phi \left(\theta_{m}^{\left(i\right)},\delta^{\left(i\right)}\right)$ are correctly classified within a certain error margin *ξ_i_*.

Given the solution of (3), a predictor (SV classifier) for the decision process *δ *= *δ*(*θ*) is

(4) $$\hat{\delta }\left(\theta_{m}\right)=\text{sign}\left(w^{\text{T}}\Phi \left(\theta_{m}\right)+b\right).$$

Assuming that the training set $S_{\delta }$ is large, the predictive power of this predictor will be high — meaning that $\hat{\delta }\left(\theta_{m}^{\left(i\right)}\right)=\delta \left(\theta^{\left(i\right)}\right)$ for most *θ*^(*i*) ^~ Θ(*θ*) — if and only if the selected markers *θ***_m _**are informative. This allows the quantitative assessment of the informativeness of the markers *θ***_m _**using the SV classifier.

Therefore, a second sample $S_{\delta }^{\prime }$ is computed which was not used to train the SV classifier, avoiding overfitting. For this sample, the predictor ${\hat{\delta }}^{\left(i\right)}=\hat{\delta }\left(\theta_{m}^{\left(i\right)}\right)$ is evaluated. These results are used to calculate the percentage of true positive classifications TP $\left(\delta^{\left(i\right)}=1\wedge {\hat{\delta }}^{\left(i\right)}=1\right)$ and false positive classifications FP $\left(\delta^{\left(i\right)}=0\wedge {\hat{\delta }}^{\left(i\right)}=1\right)$ achieved by the SV classifier. TP and FP provide information about the predictability of the outcome for *θ*^(*i*) ^using solely $\theta_{m}^{\left(i\right)}$. Thus, the marker quality can be assessed via TP and FP. If a low-dimensional **m **exists that provides TP ≈ 1 and FP ≈ 0, the parameters *θ***_m _**dominate the decision process and are good markers. For a quantification of this effect, the classification performance can be analyzed in receiver-operating characteristic (ROC) space [28].

#### 2.4.2 SV regression

Similar to the assessment of the predictive power of marker combinations for qualitative decisions, also quantitative properties may be analyzed. Therefore, we employ SV regression which allows us to compute a data-based predictor

(5) $$\hat{\varphi }\left(\theta_{m}\right)=w^{\text{T}}\Phi \left(\theta_{m}\right)+b,$$

for the quantitative property *φ *= *φ *(*θ*). To compute the nonlinear predictor, a kernel $\Phi :{\mathbb{R}}^{r}\to {\mathbb{R}}^{r^{*}}$[29] is chosen and an optimization criterion selected. In this work, we use an *ε*-insensitive loss function [30], meaning that residuals $\varphi^{\left(i\right)}-\hat{\varphi }\left(\theta_{m}^{\left(i\right)}\right)$ with an absolute value below *ε *are not penalized while larger residuals are penalized linearly. This loss function is frequently used in the literature (see, e.g., [20,30]) and results for the sample $S_{\varphi }$ in the optimization problem:

(6) $$\begin{array}{c} \underset{w,b,\xi ,\xi^{*}}{\text{minimize}}\;\frac{1}{2}w^{\text{T}}w+C\overset{N}{\underset{i=1}{\sum }}\left(\xi_{j}+\xi_{i}^{*}\right)\\ \text{subject to }\varphi^{\left(i\right)}-w^{\text{T}}\Phi \left(\theta_{m}^{\left(i\right)}\right)-b\geq \varepsilon +\xi_{i},\;i=1,\ldots ,N,\\ \;\;\;\;\;-\varphi^{\left(i\right)}+w^{\text{T}}\Phi \left(\theta_{m}^{\left(i\right)}\right)+b\geq \varepsilon +\xi_{i}^{*},i=1,\;\ldots ,\;N,\\ \;\;\;\;\;\;\;\;\;\;\;\;\;\;\xi_{i},\xi_{i}^{*}\geq 0,\;i=1,\;\ldots ,\;N. \end{array}$$

Aside from the penalization of prediction error, $\sum_{i=1}^{N}\left(\xi_{i}+\xi_{i}^{*}\right)$, flatness and a unique solution is ensured using $\frac{1}{2}w^{\text{T}}w$. The trade-off between those two is determined by the constant *C *> 0.

The optimal solution of (6) for *w *and *b *provides the optimal predictor (5) with respect to the loss function and kernel. This predictor ${\hat{\varphi }}^{\left(i\right)}=\hat{\varphi }\left(\theta_{m}^{\left(i\right)}\right)$ is applied to a second sample $S_{\varphi }^{\prime }$ to compute ${\hat{\varphi }}^{\left(i\right)}$, a prediction for *φ*^(*i*)^. Employing *φ*^(*i*) ^and ${\hat{\varphi }}^{\left(i\right)}$ the marker combination **m **might be evaluated based on the relative prediction errors, $e_{m}^{\left(i\right)}=\left|\frac{\varphi^{\left(i\right)}-\hat{\varphi }\left(\theta_{m}^{\left(i\right)}\right)}{\varphi^{\left(i\right)}}\right|$. Using $e_{m}^{\left(i\right)}$, the prediction powers of different marker combinations can be assessed and compared using, e.g., the mean error $\frac{1}{N}\sum_{i=1}^{N}e_{m}^{\left(i\right)}$. If the mean prediction error achieved by a marker combination is small, the parameters $\theta_{m}^{\left(i\right)}$ carry most of the information about *φ*^(*i*)^, and hence are suitable markers. In some situations, the information about the mean prediction error may be complemented by detailed information about the error statistics, ${\left\{e_{m}^{\left(i\right)}\right\}}_{i=1}^{N}$. These statistics may be visualized using, for instance, box plots or histograms, and provide additional insight, e.g., in the structure of the error (short- vs. long-tailed distributions) and the potential causes.

Note that the performance and predictive power of SV machines strongly depend on the available training set. For the analysis performed, we ensured that the training sets are large enough and that a further increase in its size does not result in a significant improvement of the predictors. This is, in most situations where SV machines and SV regressions are used, impossible for data analysis, as the measurement devices are limited. However, in this work we study the problem of model analysis. The size of the dataset can be increased arbitrarily by repeated simulation of the model. Besides the size of the dataset, the parameters of the SV classification and SV regression are tuned to allow for a fair comparison between the marker combinations. With this and the existence of sophisticated SV machine toolboxes (e.g., LIBSVM [31]), the observed difference between marker combinations can be assumed to be due to the predictive power of the markers.

Summing up, SV machines allow for the derivation of predictors for qualitative and quantitative properties. These predictors can be used to assess the information content of a subset **m **of the parameters about the respective properties, thereby facilitating the assessment of a quantitative evaluation of the predictive power of *θ***_m_**. For further details about SV machines we refer to [18-20,30,31] and references therein.

## 3 Results

### 3.1 Model for heterogeneous cancer cell population

To illustrate the proposed visual analytics framework, a model of the proapoptotic signaling is analyzed. Proapoptotic signaling is involved in the process of apoptosis [32-34], also called programmed cell death. Apoptosis is an important physiological process to remove infected, malfunctioning, or no longer needed cells from a multicellular organism. The apoptotic signaling pathways converge at the caspase cascade [32], where initiator caspases (e.g., caspase 8) and effector caspases (e.g., caspase 3) are activated. If the activity of effector caspases exceeds a certain threshold, apoptosis is induced.

**Figure 4:**
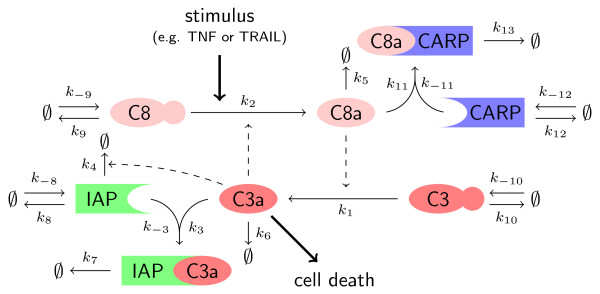
**Caspase cascade**. Illustration of proapoptotic signaling pathway [35]. Normal arrows refer to conversion reactions, dashed arrows indicate enzymatic activity, and thick arrows illustrate inputs and outputs of the system.

A variety of single cell and cell population models have been proposed to describe cellular apoptosis (see, e.g., [4-6,34-40] and references therein). In this study, we consider the mathematical model of the signal transduction which is depicted in Figure 4. This single cell model [35] is among the most cited ones. For details about the model, we refer to the original publication [35]. As the process of apoptosis induction is known to be heterogeneous, we extend the single cell model [35] by accounting for cell-to-cell variability. This is achieved by introducing differences in parameter values and initial conditions:

• From flow cytometric experiments, it is known that the amount of caspase 8 (C8), caspase 3 (C3), caspases 8- and 10-associated RING protein (CARP), and inhibitor of apoptosis protein (IAP) is different among individual cells. The differences are modeled by differences in synthesis rates (*k*_-8_, *k*_-9_, *k*_-10_, and *k*_-12_) among individual cells. The distribution of *k*_-8_, *k*_-9_, *k*_-10_, and *k*_-12 _within the population is modeled as log-normal distribution, with mean as published by Eissing et al. [35] and a coefficient of variation of 0.4 (own unpublished data). The initial conditions of C8, C3, CARP, and IAP are set to their steady state values.

• Similar to the original publication [35], the activation of the caspase cascade is modeled by a non-zero initial condition of active caspase 8, C8a(0). In the population, C8a(0) is log-normally distributed with a median of 4,000 molecules per cells and a coefficient of variation of 0.4. The variation of C8a(0) accounts for variability up-stream of the caspase cascade.

The binding affinities and kinetic rates are the same for all cells. For the numerical values, we refer to the article of Eissing et al. [35].

Given this model of the heterogeneous cell population, we analyzed (i) how the decision whether or not a cell undergoes apoptosis during the first 12 hours and (ii) how the time of cell death *T_d _*is influenced by the cell's parameters *θ *= [C8a(0), *k*_-8_, *k*_-9_, *k*_-10_, *k*_-12_]^T^. This yields two variables of interest: *δ *(= +1 ⇒ cell survived; = -1 ⇒ cell died) providing the outcome of the decision process; and *φ *(= *T_d_*) providing the time of apoptosis commitment. As indicator for apoptosis, the amount of active caspase 3 (C3a) is used. If more than 5,000 copies of C3a are present in a cell, this cell is assumed to undergo apoptosis within 10 minutes, defining the time of cell death *T_d_*. The functionals associated to the considered *δ *and *φ *are similar to (1) and (2), respectively. In the remainder, we search for a lower-dimensional subset of the parameters *θ *which provide good markers for cell death and survival as well as the time of cell death.

### 3.2 Parallel-coordinates plot establishes importance of C3 and IAP concentration for cell fate decision

**Figure 5:**
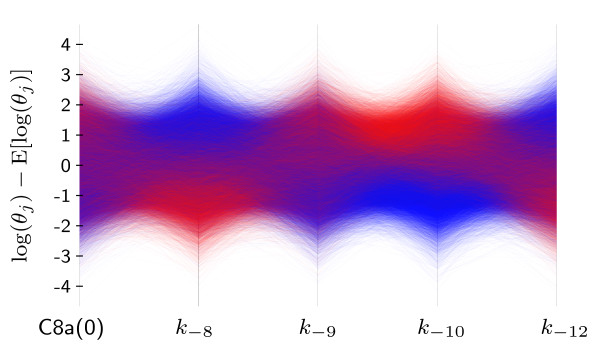
**Separation of subgroups in parallel-coordinates**. Parallel-coordinates density plot in which each polyline represents the parameter of a single cell, *θ*^(*i*)^. The color of a polyline encodes whether the cell survived (blue line) or died (red line). In order to emphasize dense regions, alpha blending with *α *= 0.03 was used for all lines. The parameters *k*_-8 _and *k*_-10 _show the best separation of colors and hence correspond to potential markers.

To study the life-death-decision, a sample $S_{\delta }$ with 100,000 members is visualized in parallel-coordinates (Figure 5). As only two classes (dead and alive) are considered, alpha blending can be used to visualize the density of each class as well as the density at the overlapping regions, where the transparent red color, representing dead cells, and the transparent blue color, representing living cells, are blended wit *α *= 0.03. Using this coloring, high-density regions appear more saturated for the individual classes and darker at their overlap.

From Figure 5, it is apparent that the second and fourth parameters (*θ***_m _**= [*k*_-8_, *k*_-10_]^T^) provide a reasonable separation between the classes (red = dead, blue = alive). Most of the surviving cells have high values of *k*_-8 _and low values of *k*_-10_, which corresponds to a high IAP expression and a low C3 expression, respectively. Although the other parameters also influence the process, their influence seems to be minor.

### 3.3 SV classification proves that C3 and IAP expression are the best markers for the cell fate decision

**Figure 6:**
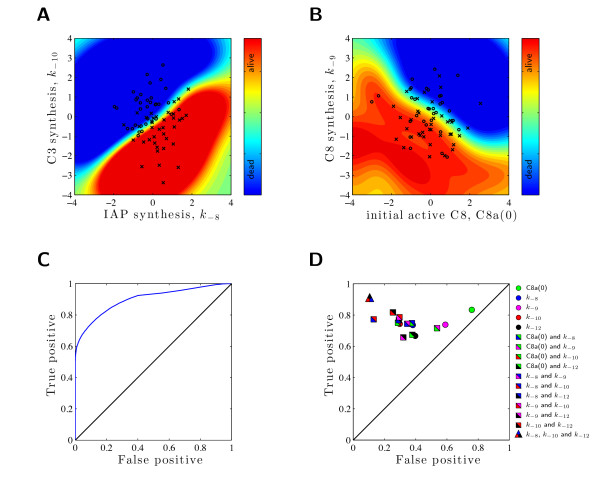
**Performance of different marker combinations**. Evaluation of classification performance using different marker combinations. **(A) **and **(B) **illustrate the classification obtained using to different two marker combinations. The prediction of the classifier (trained using 10.000 simulations) is provided as background color (blue square = alive; red square = dead) together with a test sample (^× ^= alive; ◦ = dead), which has not been used for training. **(A) **Classification employing C3 synthesis, *k*_-10_, and IAP synthesis, *k*_-8_, as markers. For the classification of cell survival: TP = 0.77, FP = 0.13. **(B) **Classification employing initial amount of C8a, C8a(0), and C8 synthesis, *k*_-8_, as markers. For the classification of cell survival: TP = 0.68, FP = 0.53. **(C) **ROC curve obtained when using C3 synthesis, *k*_-10_, and IAP synthesis, *k*_-8 _as marker. **(D) **The performance of all individual markers, all marker pairs, and the best marker triplet in ROC space. Note that an optimal classifier would be in the upper left corner.

Given the results of the visual analysis, we consider *θ***_m _**= *k*_-8_, *θ***_m _**= *k*_-10_, as well as *θ***_m _**= [*k*_-8_, *k*_-10_]^T ^and compute the classification quality using SV machines (for details see "Methods"). As can be seen in Figure 6A, the predictive power of the individual parameters is limited (*θ***_m _**= *k*_-8_: TP = 0.73, FP = 0.38; *θ***_m _**= *k*_-10_: TP = 0.74, FP = 0.29), while both markers together yield a reasonable classification performance (TP = 0.77, FP = 0.13). The corresponding ROC curve is depicted in Figure 6C and the visualization of TP and FP is provided in Figure 6D. For comparison, the alternative combinations of two markers are evaluated in terms of the area under the ROC curve (Table 1) and the TP/FP (Figure 6C).

**Table 1:** Area under the ROC curve for different marker combinations

	C8a(0)	*k*_-8_	*k*_-9_	*k*_-10_	*k*_-12_
**C8a(0)**	0.569	0.747	0.626	0.808	0.690
***k*_-8_**	0.747	**0.736**	0.760	**0.898**	0.800
***k*_-9_**	0.626	0.760	0.603	0.822	0.709
***k*_-10_**	0.808	**0.898**	0.822	**0.795**	0.858
***k*_-12_**	0.690	0.800	0.709	0.858	0.676

A common performance measure for classifiers is the area under the ROC curve. The worst and best achievable performance is 0.5 and 1, respectively. The evaluation of the area under the ROC curve verifies that if only one marker can be used, *k*_-10 _(area = 0.795) and *k*_-8 _(area = 0.736) are the best choice (on-diagonal bold numbers). In case of two markers, the combination of *k*_-8 _- *k*_-10 _allows for the best classification (area = 0.898) (off-diagonal bold numbers). For the three markers *k*_-8_, *k*_-10_, and *k*_-12_, the area under the ROC curve is 0.966.

The markers *θ***_m _**= *k*_-8 _and *θ***_m _**= *k*_-10 _outperform all other single markers and marker pairs. In addition, the marker vector *θ***_m _**= [*k*_-8_, *k*_-10_]^T ^outperforms all other combinations in terms of the area under the ROC curve. Some other combinations result in more than 50% false positive classifications (see Figure 6B). Of course, extending the marker vector, e.g., by adding *k*_-12_, results in further improvement.

### 3.4 Parallel-coordinates plots show a complex dependency of the time of death on the parameters

After the analysis of the decision process, we study the dependency of time of cell death *T_d _*on the parameters. The time of cell death *T_d _*is a quantitative property and can take any positive value, therefore an alternative visualization has to be used. One approach would be to use a different color for each line in parallel-coordinates, depending on $T_{d}^{\left(i\right)}$. Unfortunately, this approach suffers from heavy overplotting, which is why the data was split into three classes and separate plots were created for each class.

**Figure 7:**
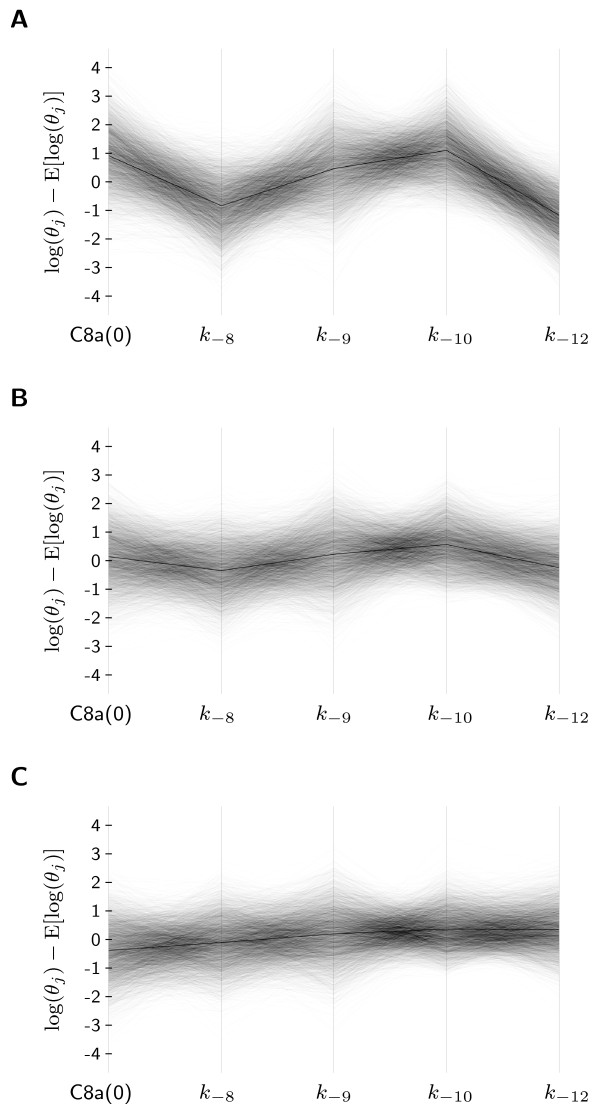
**Dependency of T_d _on the single cell parameters**. Visualization of three subsets of the sample $S_{\varphi }$ using parallel-coordinates. The subplots depict the parameter vectors of the cells having $T_{d}^{\left(i\right)}$ value: **(A) **below the 10th percentile; **(B) **between 45th percentile and the 55th percentile; and **(C) **above the 90th percentile of the *T_d _*values. The thin black lines are elements of the sample and the thicker line is the mean. A comparison of the subplots **(A) **and **(C) **shows that cells dying early and cells dying late mainly differ in the parameters C8a(0), *k*_-10_, and *k*_-12 _but also the other parameters show small offsets in one or the other way. The joint consideration of all subplots reveals that in parallel-coordinates the parameter regions, associate to different times of cell death, overlap. This indicates that several parameters will determine $T_{d}^{\left(i\right)}$.

Figure 7A-C visualize the parameter distribution in different percentile intervals for *T_d_*. A comparison of Figure 6A, visualizing the cells that die early (0 to 10th percentile), and Figure 7C, depicting the cells that die late (90 to 100th percentile), unravels o sets in all parameter dimensions. The differences are particularly prominent for C8a(0), *k*_-10_, and *k*_-12_, showing that the abundance of C3 also plays an important role in determining whether cells die early or late. Unfortunately, a closer look at Figure 7 also reveals that the parameter distributions associated to cells that undergo apoptosis at early, intermediate, and late time points strongly overlap in parallel-coordinates. This indicates that *T_d _*may depends on all parameters. Therefore, a reliable prediction of *T_d _*using only a few parameters might be infeasible.

### 3.5 SV regression reveals ubiquitous importance of IAP an C3 expression levels

To quantify the predictive power of different marker combinations with respect to *T_d_*, we employ the SV regression based approach introduced in "Methods". As a performance measure, the relative prediction error $\left|\frac{T_{d}^{\left(i\right)}-{\hat{T}}_{d}^{\left(i\right)}}{T_{d}^{\left(i\right)}}\right|$, their ${\hat{T}}_{d}^{\left(i\right)}$ is the prediction of the SV machine. Details on the implementation may be found in "Methods".

**Figure 8:**
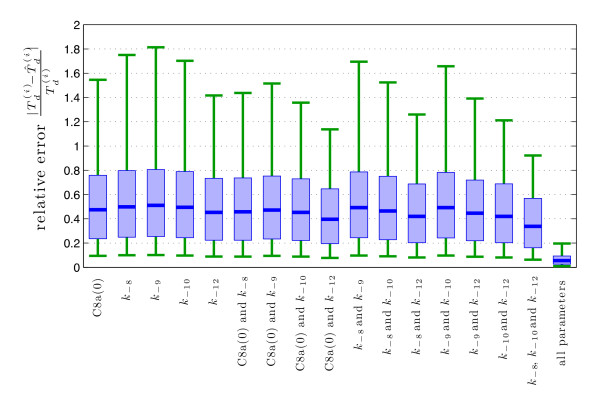
**SV regression analysis of T_d_**. Statistical analysis of the predictive power of SV machines using different markers **m**. Shown are box plots for the relative prediction error: median (blue line), interquartile range (light blue area), and 10th to 90th percentile range (green line). The combination of two markers which results in the smallest median prediction error is (C8a(0) and *k*_-12_), but all over combinations achieve prediction errors which are alike. The last column shows that information about all parameter enables extremely precise predictions using SV machines.

At first, we study the potential combinations of two markers proposed by the parallel-coordinates plots: *k*_-10 _and *k*_-12_; C8a(0) and *k*_-10_; and C8a(0) and *k*_-12_. Out of those, the best performance with a median prediction error of 40% is achieved by C8a(0) and *k*_-12_, which also outperforms all other combinations of two markers. Interestingly, all marker combinations achieve a median prediction error between 40 and 50%, as shown in Figure 8. This illustrates two things: On the one hand, markers allowing for a distinction between early and late dying cells do not necessarily enable a good prediction of the death time *T_d_*, as here also the cells dying in an intermediate interval dominate the statistic. On the other hand, this quantification proves that even the best combination of two markers provides only very limited predictive power. Thus, unlike the decision which predominantly depends an C3 and IAP expression, the time of cell death is highly sensitive to changes in all parameters.

## 4 Conclusion

### 4.1 Visual analytics enable an in-depth analysis of complex population models

In this article, a novel explorative approach has been presented to determine markers for decision processes in heterogeneous populations. It has been shown that methods used for data analysis can also be employed to gain insight into complex models, where common analytical methods seem to reach their limits. Especially, the potential of parallel-coordinates plots and support vector machines has been illustrated. While the first allows for the study of large, high-dimensional datasets and the selection of potential markers, the latter can provide a quantitative assessment of their predictive power. Using both methods, the source of qualitative and quantitative cell-to-cell variability may be unraveled.

This article provides a case study evaluating the potential of combining visualization and automated methods for the assessment of complex system models. The considered system class is only one example and the proposed framework can be generalized easily to other systems and questions.

### 4.2 Analysis of heterogeneous cell population allows for novel insight

We have illustrated the proposed visual analytics approach by analyzing a cell population model for proapoptotic signaling, which plays an essential role in programmed cell death. We have studied the cell fate decision as well as the time of cell death. These properties were analyzed before (see, e.g., [5]) in a purely qualitative way and without the tools proposed in this work.

Our study shows that parallel-coordinates plots are a proper tools to determine potential markers. The predictive power of these markers can then be quantified using SV machines. In this study, the markers we found agree well with those found in the literature. In particular, the important role of IAP—also called XIAP—for cell death commitment is outlined in several publications [39,41]. While C3 abundance is known to be important [39], our analysis suggests that the amount of available C3 could be even more important than expected.

In addition, our analysis indicates that, under normal conditions, the time of cell death strongly depends on all parameters, which has been hypothesized earlier [5]. Only under altered conditions, e.g., a strongly increased initial amount of C8a(0), some parameters become more important than others (results not shown). This is again in agreement with the results of Spencer et al. [5]. Furthermore, this finding of a varying importance of parameters depending on the experimental setup, provides hints for possible future experiments. Thus, our visual analytics approach we propose also provides helpful feedback for model validation and development.

### 4.3 Outlook

In this work, we have proposed a method to determine decision markers for given models. However, all model possess uncertainties, rendering an uncertainty-aware analysis crucial. Therefore, a workflow including model development, parameter estimation, uncertainty analysis, and marker prediction has to be established. This requires improved modeling and parameter estimation tools, as well as methods to evaluate the uncertainty of the marker prediction, arising from model uncertainties.

Given such a workflow, beyond the analysis of models, our analysis tools might also be used to guide the search for biomarkers. This is possible as our methods allows for the assessment of the importance of any parameters which are different among cells of the population. Among others, the importance of common biomarkes, e.g., expression levels and transcription factor/protein abundance, may be determined based on a model of the population. This is much in the same way as the target selection using sensitivity analysis of single cell models based on ordinary differential equations (see, e.g., [42]). However, the marker selection requires population models, as differences between cells have to be considered, and is therefore more challenging.

## Methods

### Software

The model of the heterogeneous cell population was implemented in MATLAB using the SBtoolbox2 [43]. For the SV classification and the SV regression, the LIBSVM toolbox for MATLAB is employed [31]. The visualization software for parallel-coordinates was implemented in C++ using the Qt library Version 4.8.0 and OpenGL.

### Numerics

For the SV classification and SV regression, we employed as kernels radial basis function with *γ *= 0.25. The SV regression parameter which defines the interval of insensitivity was set to 0.01. All remaining parameters are set to the default constants, see LIBSVM manual. To improve the performance of the SV machines, we applied a log-transformation to the parameters *θ*.

## Competing interests

The authors declare that they have no competing interests.
